# Non-invasive detection of Parkinson’s disease based on speech analysis and interpretable machine learning

**DOI:** 10.3389/fnagi.2025.1586273

**Published:** 2025-04-30

**Authors:** Huanqing Xu, Wei Xie, Mingzhen Pang, Ya Li, Luhua Jin, Fangliang Huang, Xian Shao

**Affiliations:** ^1^The School of Medical Information Engineering, Anhui University of Chinese Medicine, Hefei, China; ^2^Jiangxi Medical College, Nanchang University, Nanchang, Jiangxi, China; ^3^Division of Nephrology, National Clinical Research Center for Kidney Disease, State Key Laboratory of Organ Failure Research, Nanfang Hospital, Southern Medical University, Guangzhou, China

**Keywords:** Parkinson’s disease, speech analysis, machine learning, non-invasive, early detection

## Abstract

**Objective:**

Parkinson’s disease (PD) is a progressive neurodegenerative disorder that significantly impacts motor function and speech patterns. Early detection of PD through non-invasive methods, such as speech analysis, can improve treatment outcomes and quality of life for patients. This study aims to develop an interpretable machine learning model that uses speech recordings and acoustic features to predict PD.

**Methods:**

A dataset of speech recordings from individuals with and without PD was analyzed. The dataset includes features such as fundamental frequency (Fo), jitter, shimmer, noise-to-harmonics ratio (NHR), and non-linear dynamic complexity measures. Exploratory data analysis (EDA) was conducted to identify patterns and relationships in the data. The dataset was split into 70% training and 30% testing sets. To address class imbalance, synthetic minority oversampling technique (SMOTE) was applied. Several machine learning algorithms, including K-Nearest Neighbors (KNN), Support Vector Machine (SVM), Decision Trees, Random Forests, and Neural Networks, were implemented and evaluated. Model performance was assessed using accuracy, recall, F1 score, and area under the receiver operating characteristic curve (AUC-ROC) metrics. SHapley Additive exPlanations (SHAP) were used to explain the models and evaluate feature contributions.

**Results:**

The analysis revealed that features related to speech instability, such as jitter, shimmer, and NHR, were highly predictive of PD. Non-linear metrics, including Recurrence Plot Dimension Entropy (RPDE) and Pitch Period Entropy (PPE), also made significant contributions to the model’s predictive power. Random Forest and Gradient Boosting models achieved the highest performance, with an AUC-ROC of 0.98, recall of 0.95, ensuring minimal false negatives. SHAp values highlighted the importance of fundamental frequency variation and harmonic-to-noise ratio in distinguishing PD patients from healthy individuals.

**Conclusion:**

The developed machine learning model accurately predicts Parkinson’s disease using speech recordings, with Random Forest and Gradient Boosting algorithms demonstrating superior performance. Key predictive features include jitter, shimmer, and non-linear dynamic complexity measures. This study provides a reliable, non-invasive tool for early PD detection and underscores the potential of speech analysis in diagnosing neurodegenerative diseases.

## 1 Introduction

Parkinson’s disease (PD) is a progressive neurodegenerative disorder primarily affecting motor functions, leading to tremors, muscle rigidity, and coordination difficulties. It is one of the most common neurodegenerative diseases (Kouli et al., 2018). As the aging of population, the prevalence of PD is expected to rise, making early diagnosis and intervention crucial for improving patient prognosis. Currently, PD is diagnosed through clinical assessments such as neurological exams and imaging techniques, but these methods are limited by their reliance on observable symptoms, which may only appear after significant disease progression. Speech changes are increasingly recognized as early indicators of Parkinson’s disease, often manifesting as reduced vocal intensity, monotone speech, and impaired pitch modulation (Ciucci et al., 2013). These changes are typically caused by bradykinesia and rigidity affecting the laryngeal and respiratory muscles. Importantly, speech abnormalities may appear years before classical motor symptoms, making them valuable for early screening. In addition to speech changes, other prodromal markers of PD have been identified, including REM sleep behavior disorder (RBD), hyposmia, constipation, and subtle cognitive decline (Kang et al., 2016). Among these, RBD is currently considered one of the most specific early indicators, often preceding PD diagnosis by several years. Combining speech-based biomarkers with established non-motor indicators like RBD could enhance the sensitivity and robustness of early detection strategies. These changes can be subtle and are frequently overlooked during clinical evaluations. However, recent advances in machine learning have made it possible to develop models capable of accurately predicting the presence of PD from speech recordings (Suppa et al., 2022). This non-invasive approach offers the potential for early detection, enabling timely intervention and better management of PD.

In the realm of PD diagnosis, several studies have utilized speech features to predict the presence of the disease. For instance, researchers have analyzed speech characteristics such as jitter, shimmer, and fundamental frequency (F0) to distinguish PD patients from healthy controls (Zheng et al., 2024). Machine learning can automatically capture both linear and non-linear relationships between input features. Previous studies have applied support vector machines (SVM), Random Forests, and deep learning models, to classify PD using speech data (Hossain and Amenta, 2024; Ali et al., 2024; Moro-Velazquez et al., 2021). With advancements in feature engineering, this study applied Multinomial Naive Bayes (Multinomial NB) for dysarthria detection in PD, achieving 95% classification accuracy, demonstrating the effectiveness in high-dimensional sparse feature selection (Alshammri et al., 2023). The deep learning techniques (Meghraoui et al., 2016; Ul Haq et al., 2022; Liang et al., 2023) has significantly improved the ability to model non-linear relationships. For example, Quan et al. (2022) innovatively developed a time-domain-space-domain joint deep learning framework (TD-2DCNN + 1D-CNN) that achieved high cross-language detection accuracy in complex speech tasks (sustained vowels, short sentence reading) for Mandarin (75.3–81.6%) and Spanish (92%), and visualized the Mel-spectrogram features to reveal the key role of low-frequency regions (<2 kHz) in PD-related voice variability, providing interpretable evidence for clinical diagnosis.

These models have shown promising results, but challenges remain, including insufficient statistical validation, limited interpretability, and the risk of overfitting when training deep learning models on small sample sizes. Therefore, there is a need for more robust machine learning models to explain the complex non-linear relationships between various speech features and disease status. This study aims to bridge this gap by developing a machine learning model that could accurately predict PD using speech recordings. By analyzing features extracted from patient speech, including jitter, shimmer, and fundamental frequency, we seek to identify key biomarkers associated with PD. Ultimately, a powerful, scalable model can be established and serve as a non-invasive diagnostic tool for early PD detection, contributing to improved clinical decision-making.

## 2 Materials and methods

### 2.1 Dataset description

This study utilized a PD speech dataset, which comprises biomedical speech measurements from 31 individuals, including 23 PD patients. Each column in the dataset represents a specific speech measurement, and each row corresponds to one of the 195 speech recordings from these individuals. The “status” column indicates the health status of each individual, with healthy subjects marked as “0” and PD patients marked as “1.” The dataset is stored in ASCII CSV format, with each row representing a single speech recording instance. Each patient has approximately six speech recordings, and the individual’s name and recording number are indicated in the first column.

The dataset attributes include: Individual name and recording number; MDVP:Fo (Hz): Average fundamental frequency of the vocal cords; MDVP:Fhi (Hz): Maximum fundamental frequency of the vocal cords; MDVP:Flo (Hz): Minimum fundamental frequency of the vocal cords; MDVP:Jitter (%), MDVP:Jitter (Abs), MDVP:RAP, MDVP:PPQ, Jitter:DDP: Several measures of variation in the fundamental frequency of the vocal cords; MDVP:Shimmer, MDVP:Shimmer (dB), Shimmer:APQ3, Shimmer:APQ5, MDVP:APQ, Shimmer:DDA: Several measures of variation in the amplitude of the vocal cords; NHR, HNR: The ratio of noise to harmonic components in the speech signal; status: Health status of the individual (1 for PD patients, 0 for healthy individuals); RPDE, D2: Two non-linear dynamic complexity measures; DFA: The fractal scaling exponent of the signal; spread1, spread2, PPE: Three non-linear measures of variation in the fundamental frequency. For detailed information about the dataset, please refer to reference (Little et al., 2008; Little et al., 2007). The data collection and de-identification processes have been approved by the relevant ethics committee.

## 2.2 Data preprocessing

In the data preprocessing stage, the first step was processing missing data. Features with missing values exceeding 10% were excluded from the dataset. For other features with missing values, random forest imputation was applied. All data were standardized and normalized to ensure compatibility with the subsequent model construction.

## 2.3 Feature selection

**Figure 1:**
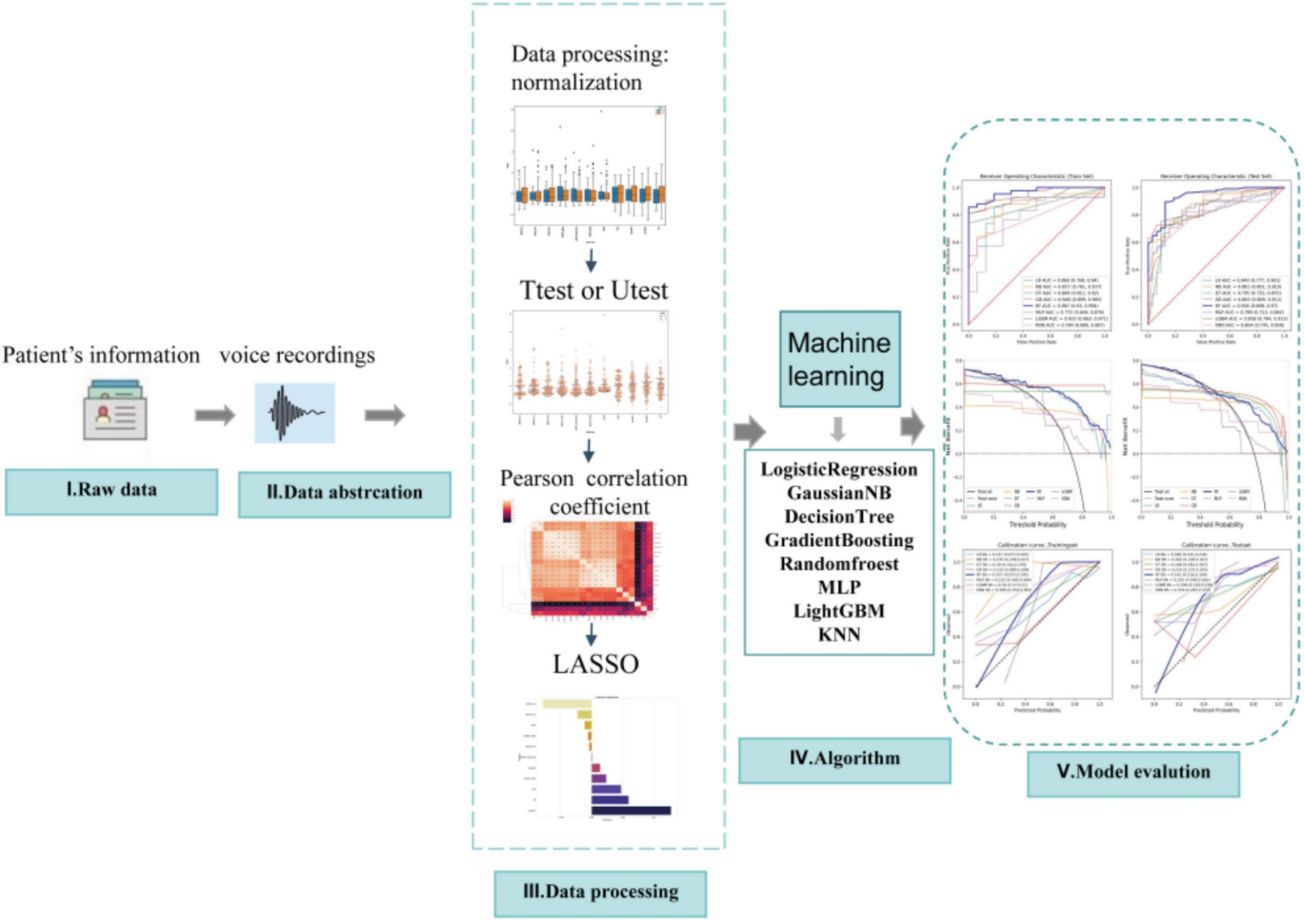
Flowchart of study.

This study included data from 195 speech recordings, with 48 healthy speech recordings (labeled as 0) and 147 PD speech recordings (labeled as 1). The data were randomly divided into training and validation sets in a 7:3 ratio. Feature selection and model construction were performed on the training set, while the validation set was used to test the model’s performance. The study involved a total of 22 features, and the feature selection process is illustrated in Figure 1. Initially, an independent sample *t*-test (Kim, 2015) or Mann–Whitney *U* test (McKnight and Najab, 2010) (*p* < 0.05) was used to perform preliminary selection of differentially significant features. Subsequently, the max-min normalization method (Mazziotta and Pareto, 2022) was applied to the selected features to eliminate the influence of units and dimensions, ensuring the reliability of the results. Next, Pearson correlation analysis (Cohen et al., 2009) was used to further refine the features. The LASSO (Least Absolute Shrinkage and Selection Operator) (Ranstam and Cook, 2018) regression method was then used for dimensionality reduction. LASSO reduces all regression coefficients to zero by adjusting the weight *λ*, setting the coefficients of many irrelevant features to zero. The optimal λ value was determined by 10-fold cross-validation to minimize the cross-validation error. The non-zero features were used to fit the regression model and were combined into the optimal set of imaging features. The scikit-learn package (Kramer and Kramer, 2016) was used to build the LASSO regression model.

## 2.4 Model construction and training

Based on the selected features, eight commonly used machine learning algorithms were employed to construct the predictive model, including Naive Bayes, Logistic Regression, Decision Trees, Random Forest, Gradient Boosting Machine (GBM), Support Vector Machines (SVM), Multi-layer Perceptron (MLP), and LightGBM. During model training, 10-fold cross-validation was used to evaluate the stability and performance of the models, and grid search was applied to optimize the hyperparameters of each model.

## 2.5 Evaluation metrics

The classification performance of the models was assessed using accuracy, sensitivity, specificity, F1 score, positive predictive value, and negative predictive value. Additionally, ROC curves, calibration curves, and decision curves were generated to provide a more comprehensive evaluation of the model’s performance. SHapley Additive exPlanations (SHAP) were used to explain the models and evaluate feature contributions. The independent variables in the dataset were measured using various units (such as Hz, dB, %, and absolute values), resulting in significant variations in data units and considerable disparities between feature values. To address this issue, a z-score data scaling technique was employed to standardize the measurements.

## 2.6 Statistical analysis

Data extraction and management for this study were performed using the Scipy.stats package (Gommers et al., 2024) in Python. For each feature, the Shapiro–Wilk test was first used to assess its normality of distribution. For features that did not follow a normal distribution, the Mann–Whitney *U* test was used to evaluate their correlation with the target variable. For normally distributed features, Levene’s test was applied to assess variance homogeneity, and based on the variance properties, either the Student’s *t*-test or Welch’s *t*-test was used for further analysis.

Additionally, 1,000 bootstrap resampling iterations were used to calculate confidence intervals for AUC values and Brier scores. The binary classification threshold for the predicted probabilities was determined by the maximum Youden index in the training set. All statistical tests were conducted with a two-tailed *p*-value of less than 0.05 considered statistically significant.

## 3 Results

### 3.1 Characteristics of study participants

A total of 195 speech recording were included in the study. The training set consisted of 137 voice recording, while the test set included 58 voice recording. Detailed statistics of the clinical characteristics are presented in Table 1.

**Table 1:** Statistical information of clinical characteristics.

Feature	Non-Parkinson’s disease (*N* = 48)	Parkinson’s disease (*N* = 147)	Coefficient	Standard Error	z	*P-*value
MDVP_Fo_Hz	181.938 ± 52.179	145.181 ± 32.238	−0.0221	0.004	−4.960	0.000
MDVP_Fhi_Hz	223.637 ± 95.714	188.441 ± 88.038	−0.0037	0.002	−2.212	0.027
MDVP_Flo_Hz	145.207 ± 58.142	106.894 ± 32.164	−0.0191	0.004	−4.845	<0.001
MDVP_Jitter	0.004 ± 0.002	0.007 ± 0.005	462.128	107.799	4.287	<0.001
MDVP_RAP	0.002 ± 0.001	0.004 ± 0.003	810.971	192.905	4.204	<0.001
MDVP_PPQ	0.002 ± 0.001	0.004 ± 0.003	937.909	210.070	4.465	<0.001
Jitter_DDP	0.006 ± 0.003	0.011 ± 0.01	270.457	64.323	4.205	<0.001
MDVP_Shimmer	0.018 ± 0.005	0.034 ± 0.02	119.999	25.989	4.617	<0.001
MDVP_Shimmer_dB	0.163 ± 0.057	0.321 ± 0.207	12.353	2.681	4.607	<0.001
Shimmer_APQ3	0.01 ± 0.003	0.018 ± 0.011	177.112	39.744	4.456	<0.001
Shimmer_APQ5	0.011 ± 0.003	0.02 ± 0.013	189.555	42.553	4.455	<0.001
MDVP_APQ	0.013 ± 0.004	0.028 ± 0.018	206.004	42.223	4.879	<0.001
Shimmer_DDA	0.029 ± 0.01	0.053 ± 0.032	59.034	13.247	4.456	<0.001
NHR	0.011 ± 0.019	0.029 ± 0.044	39.860	14.879	2.679	0.007
HNR	24.679 ± 3.399	20.974 ± 4.324	−0.258	0.055	−4.658	<0.001
RPDE	0.443 ± 0.091	0.517 ± 0.101	7.406	1.806	4.101	<0.001
DFA	0.696 ± 0.051	0.725 ± 0.055	10.233	3.248	3.150	0.002
spread1	−6.759 ± 0.636	−5.333 ± 0.967	2.397	0.373	6.417	<0.001
spread2	0.16 ± 0.062	0.248 ± 0.078	17.535	3.157	5.555	<0.001
D2	2.154 ± 0.307	2.456 ± 0.374	2.619	0.587	4.458	<0.001
PPE	0.123 ± 0.044	0.234 ± 0.084	32.291	5.182	6.231	<0.001

The analysis revealed significant correlations between variables such as ‘MDVP_APQ,’ ‘MDVP_Shimmer,’ ‘DFA,’ ‘spread1,’ ‘D2,’ ‘MDVP_Jitter,’ ‘NHR,’ ‘spread2,’ ‘MDVP_Fhi,’ ‘MDVP_Fo,’ and ‘MDVP_Flo,’ indicating that these variables are highly correlated and represent different measurements of the same underlying attributes. These findings align with the descriptive data, supporting the notion that these variables reflect the same fundamental properties. The analysis also showed that PD patients showed significantly higher MDVP_APQ (0.028 ± 0.018), MDVP_Shimmer (0.034 ± 0.02), DFA (0.725 ± 0.055), spread1 (−5.333 ± 0.967), D2 (2.456 ± 0.374), MDVP_Jitter (0.007 ± 0.005), NHR (0.029 ± 0.044), and spread2 (0.248 ± 0.078).

### 3.2 Feature selection

**Figure 2:**
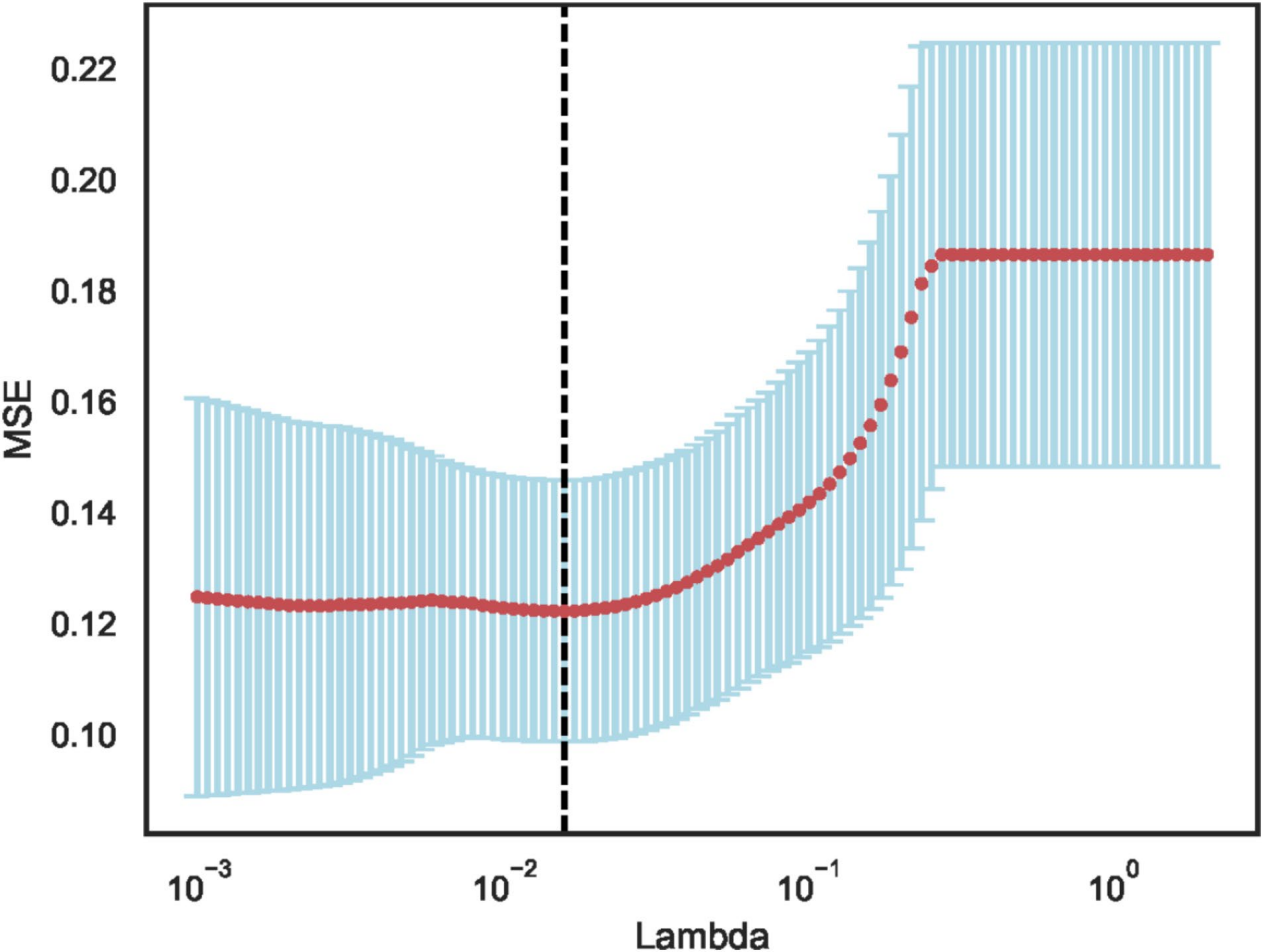
Model deviation versus parameter variation curve.

**Figure 3:**
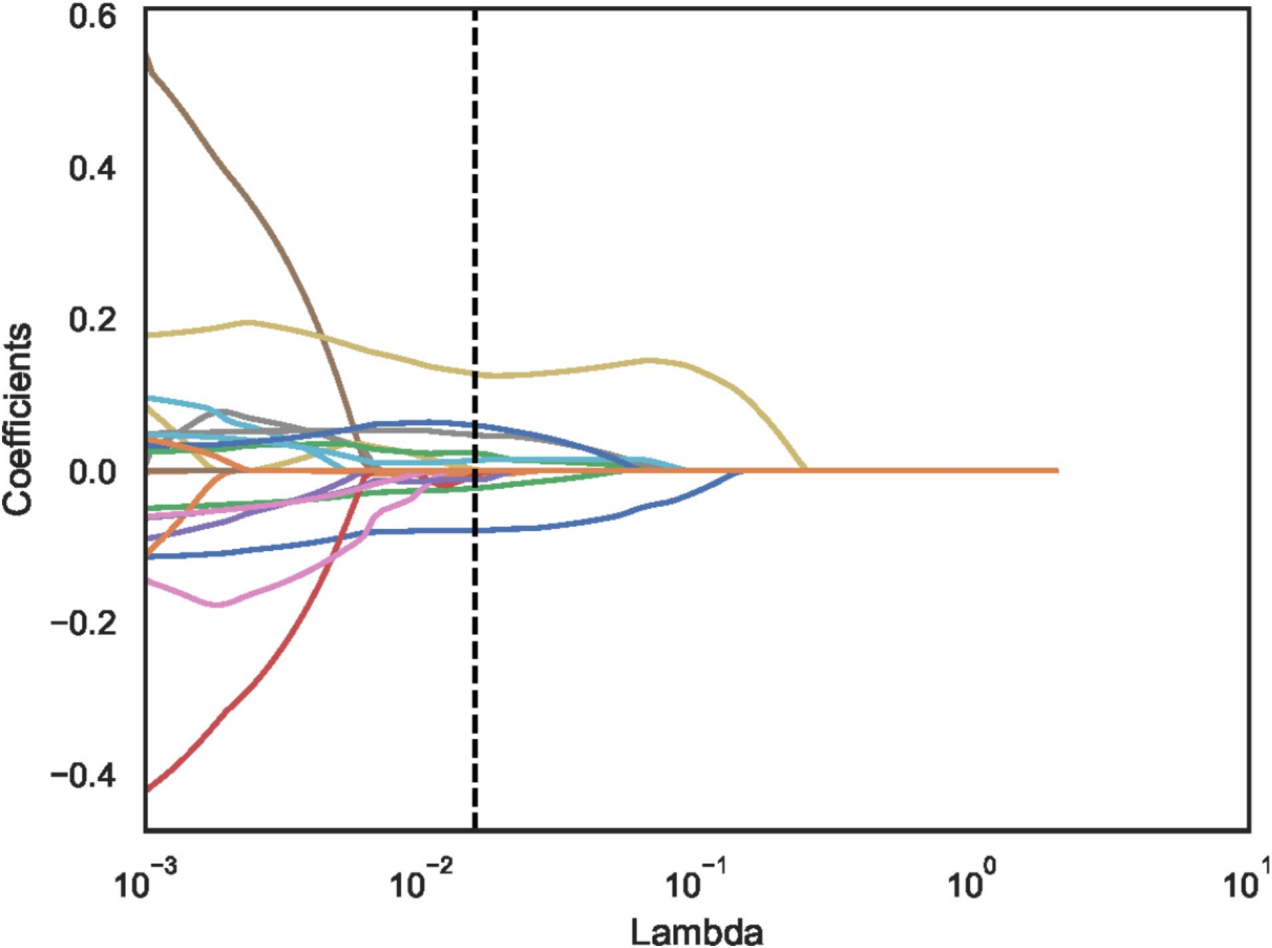
Least absolute shrinkage and selection operator (LASSO) convergence coefficient diagram with the curve of feature coefficients changing with log (*λ*).

**Figure 4:**
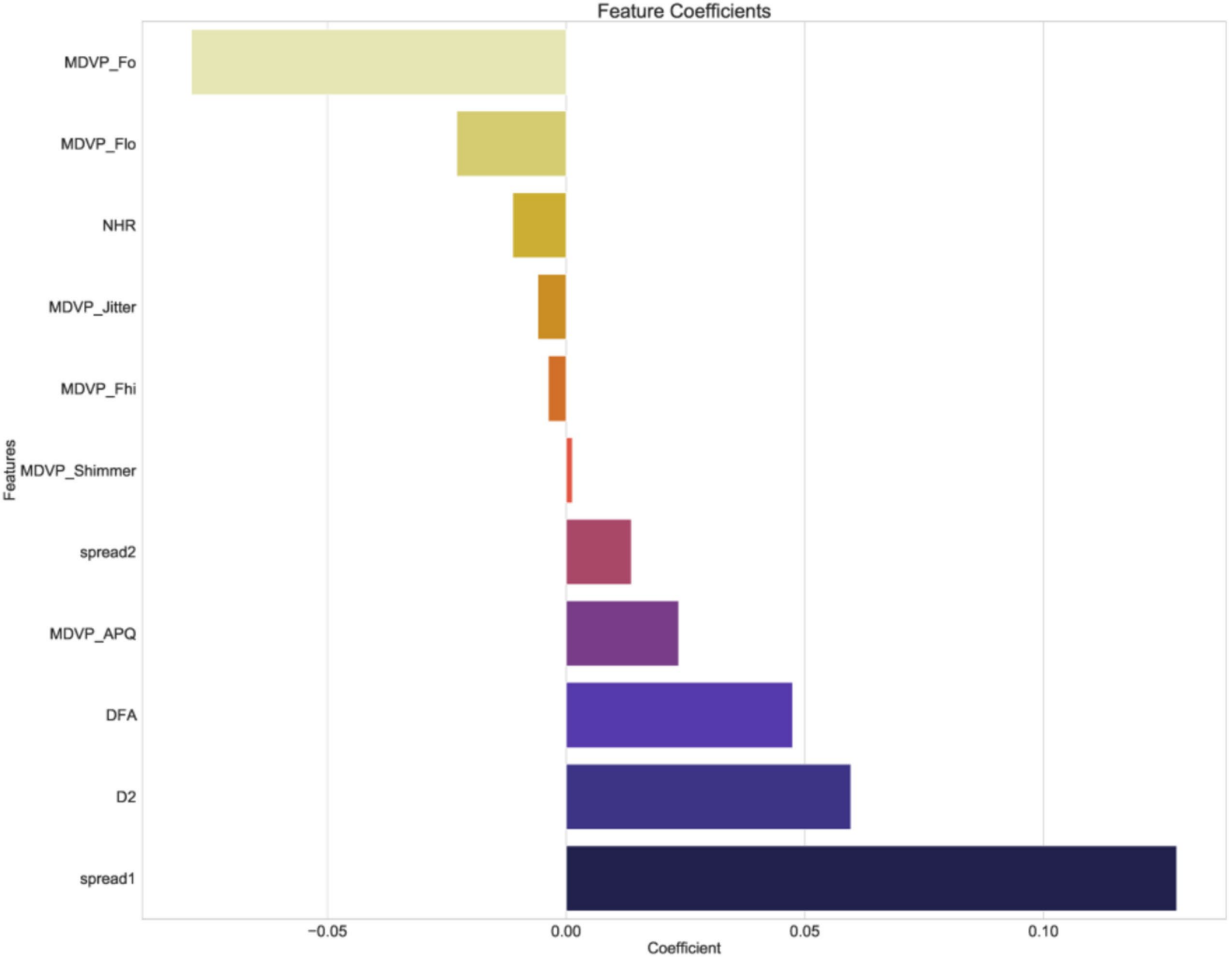
The coefficients of screened features.

Using independent sample t-test or Mann–Whitney *U* test, the number of features was reduced from 22 to 21 (*p* < 0.05). Pearson correlation analysis resulted in 14 imaging features. The LASSO regression method was then used to select the optimal 11 features. When the LASSO *λ* value was set to 0.015625, the optimal model was achieved (Figures 2–4).

The dotted line indicates the optimal log (*λ*) = 0.015625 value and the location of 1 standard error.

The curves are generated based on the λ value of the crossover operation. Each curve represents the change trajectory of the first feature. As the penalty coefficient λ gradually increases, an increasing number of feature coefficients are compressed to zero. The dotted line indicates the 11 features selected in this study.

### 3.3 Performance of machine learning models

**Figure 5:**
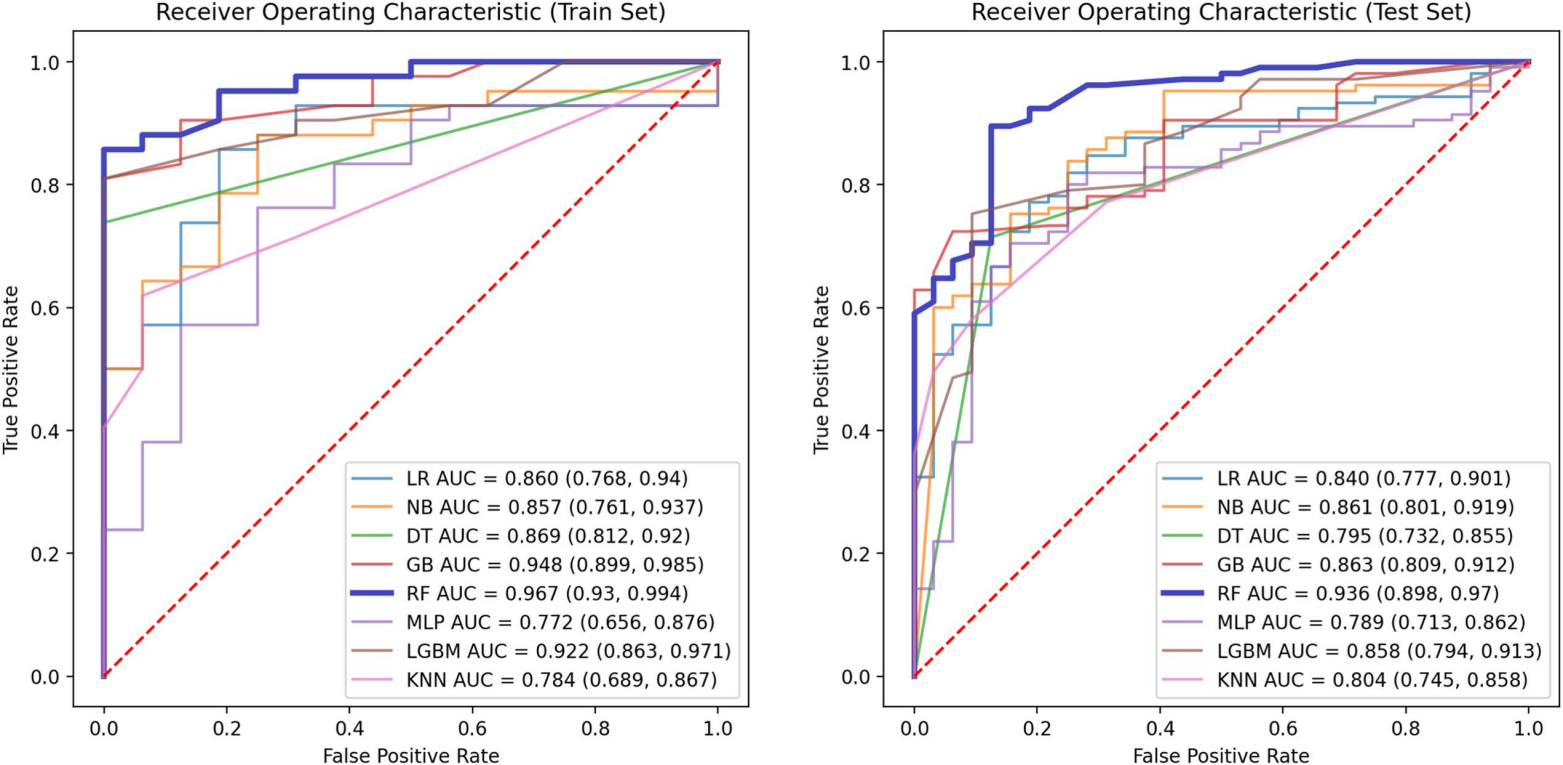
ROC curves of the training set (left) and the test set (right).

**Figure 6:**
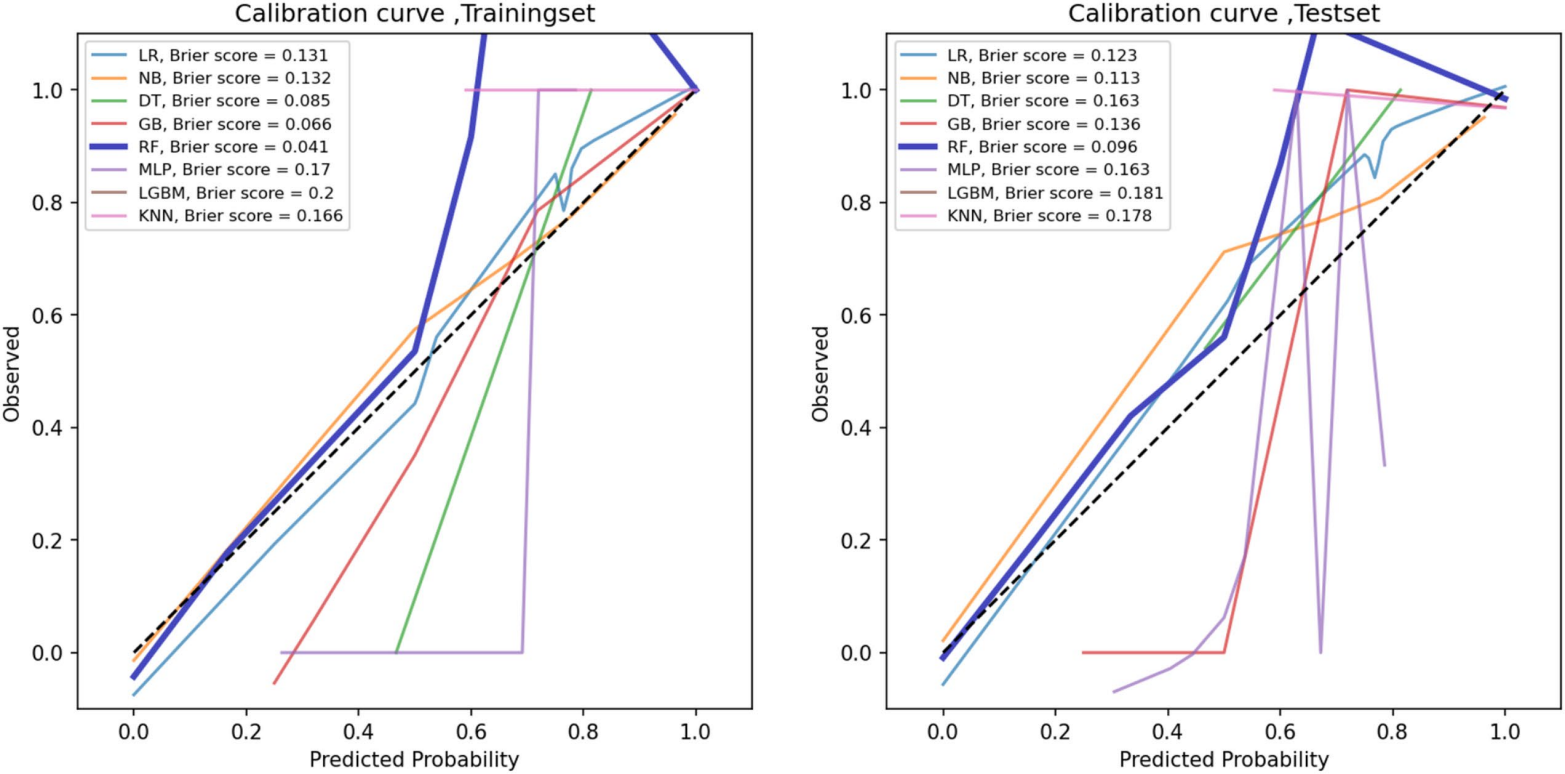
Calibration curves for the training set (left) and the test set (right).

**Figure 7:**
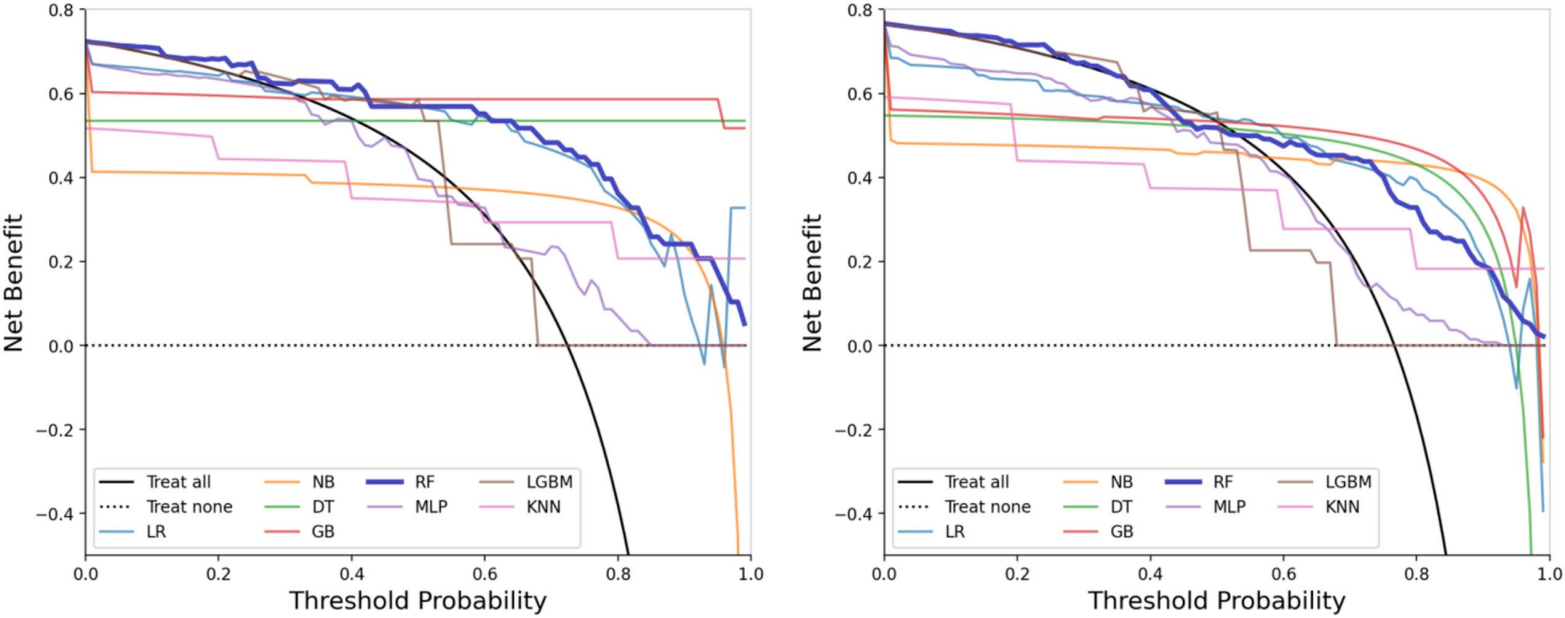
Decision curve analysis (DCA) curves of the training set (left) and the test set (right).

The performance metrics of the machine learning models are summarized in Table 2. The ROC curves are shown in Figure 5, with calibration curves in Figure 6, and decision curve analysis (DCA) in Figure 7. Notably, tree-based models (RF and LightGBM) exhibited higher AUC, outperforming other models. Specifically, the Random Forest model achieved the highest AUC of 0.936. The calibration curve showed a Brier score of 0.096 for the Random Forest model, and DCA demonstrated strong practical value in clinical decision-making.

**Table 2:** Performance of models in training and test set.

Model	AUC	Accuracy	Sensitivity/recall	Specificity	F1-score	PPV/precision	NPV	Cut-off
LR	0.860|0.840	0.845|0.774	0.881|0.781	0.750|0.750	0.892|0.841	0.902|0.911	0.706|0.511	0.500|0.500
NB	0.857|0.861	0.655|0.693	0.548|0.619	0.938|0.938	0.697|0.756	0.958|0.970	0.441|0.429	0.500|0.500
DT	0.869|0.795	0.810|0.752	0.738|0.714	1.000|0.875	0.849|0.815	1.000|0.949	0.593|0.483	0.500|0.500
GB	0.948|0.863	0.862|0.766	0.810|0.724	1.000|0.906	0.895|0.826	1.000|0.962	0.667|0.500	0.500|0.500
RF	0.967|0.936	0.872|0.852	0.886|0.875	0.967|0.906	0.898|0.863	1.000|0.986	0.640|0.483	0.500|0.500
MLP	0.772|0.789	0.672|0.715	0.643|0.676	0.750|0.844	0.740|0.785	0.871|0.934	0.444|0.443	0.500|0.500
LGBM	0.922|0.858	0.862|0.788	0.810|0.752	1.000|0.906	0.895|0.845	1.000|0.963	0.667|0.527	0.500|0.500
KNN	0.784|0.804	0.621|0.606	0.500|0.495	0.938|0.969	0.656|0.658	0.955|0.981	0.417|0.369	0.500|0.500

The left side of “|” is in the training set, and the right side is in the test set. AUC, the area under the curve; PPV, positive predictive value; NPV, Negative predictive value.|The left side of “|” is in the training set, and the right side is in the test set. AUC, the area under the curve; PPV, positive predictive value; NPV, Negative predictive value.

### 3.4 SHAP value analysis

**Figure 8:**
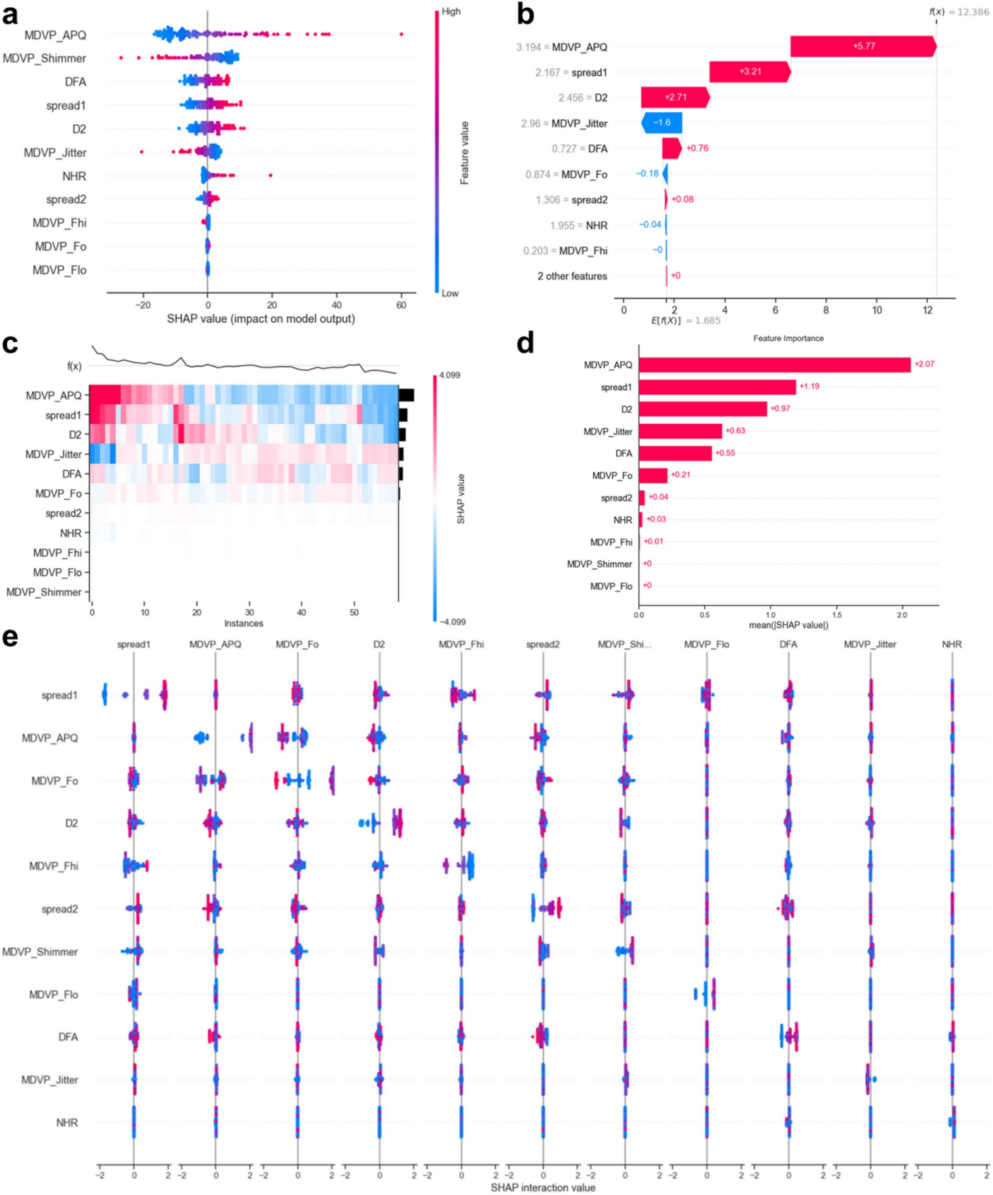
SHapley Additive exPlanations (SHAP) interpretability of random forest **(a–e)**.

Figure 8 displays the SHAP values. The results indicated that elevated values of ‘MDVP_APQ’ (Amplitude Perturbation Quotient), ‘DFA’ (Detrended Fluctuation Analysis), ‘spread1’ and ‘spread2’ (non-linear measures of frequency variation), ‘D2’ (Correlation Dimension), ‘NHR’ (Noise-to-Harmonics Ratio), and ‘MDVP_Fo’ (Average Fundamental Frequency) were associated with a higher likelihood of PD. Conversely, lower levels of ‘MDVP_Shimmer’ (Amplitude variation), ‘MDVP_Jitter’ (Frequency variation), ‘MDVP_Fhi’ (Maximum Fundamental Frequency), and ‘MDVP_Fo’ were also indicative of PD presence.

## 4 Discussion

The PD is a progressive neurodegenerative disorder that affects motor function and speech patterns. As symptoms appear, patients may experience a significant decline in quality of life. Early identification of the disease, particularly when symptoms are not yet obvious, can significantly improve treatment outcomes and slow disease progression. Early diagnosis enables timely initiation of pharmacological treatments and supportive therapies including speech and physical therapy, which are more effective when introduced in the early stages. This study developed a novel method for predicting PD using speech data and machine learning techniques, demonstrating the enormous potential of speech analysis for early diagnosis.

This study demonstrates that multiple acoustic features in speech are highly correlated with PD, especially those related to speech stability (such as jitter, shimmer, NHR) and non-linear dynamic complexity measures (RPDE, PPE), which show a significant impact on disease prediction. Tree-based models, such as Random Forest and Gradient Boosting Trees, exhibited excellent performance in our experiments, with an AUC-ROC of 0.936. This indicates that these models can accurately differentiate between PD patients and healthy individuals, with a low false-negative rate. Additionally, SHAP value analysis further revealed the core role of fundamental frequency variation and harmonic-to-noise ratio in distinguishing patients from healthy individuals, providing valuable insights for future research.

By comparing with machine learning algorithms, such as Support Vector Machine, LightGBM, and K-Nearest Neighbors, we found that Random Forest and Gradient Boosting Tree outperformed traditional statistical methods in terms of AUC values and classification performance. The RF model achieved the highest AUC value of 0.936, indicating its strong ability to handle high-dimensional and imbalanced speech data. Furthermore, tree-based models not only improved classification accuracy but also ensured model interpretability, which is crucial for practical clinical applications. Calibration curves and decision curve analysis further demonstrated that the RF model has strong clinical decision support capabilities, with a Brier score of 0.096, indicating high reliability of the predictions.

Feature selection was a key part of this study, employing methods such as t-tests, Mann–Whitney U tests, Pearson correlation analysis, and LASSO regression to successfully identify key acoustic features associated with Parkinson’s disease. Through an in-depth analysis of these features, we confirmed the importance of jitter, shimmer, fundamental frequency, and non-linear dynamic complexity measures (such as RPDE and PPE) in disease prediction. Notably, some features (such as MDVP:APQ, DFA, and NHR) have significant biological meaning in distinguishing PD patients and may reflect early acoustic changes in Parkinson’s disease, providing new biomarkers for early clinical screening.

With the development of artificial intelligence (Li et al., 2023; Saw, 2023), many studies have used AI technology to detect Parkinson’s disease. For example, Teixeira and Fernandes (2014) proposed a voice health classification framework based on Jitter, Shimmer, and HNR parameters. They found that gender differences significantly influenced the Jitter parameter in healthy populations, while different vowels (/a/, /i/, /u/) and pitch (high/low/middle) caused significant differences in multiple parameters such as Shimmer, apq3, and HNR. This work established an acoustic parameter benchmark for healthy voices, which serves as a critical baseline for pathological voice detection. Ji et al. (2025) proposed a graph-based multi-label voice feature selection algorithm that modeled Parkinson’s disease (PD) subtypes (tremor, gait freezing, swallowing disorder) as a multi-label learning task. By integrating continuous vowels (/a/) and dynamic pronunciation tasks (/pa-ka-la/) with acoustic features, they combined a graph model to select key biomarkers, achieving a 12.6% improvement in subtype joint recognition accuracy over traditional single-label methods (such as LASSO), significantly optimizing classification specificity. Ahsan et al. (2022) proposed a multi-dimensional framework for machine learning disease diagnosis through systematic bibliometric analysis (Scopus/WOS database, 1,216 papers). They reviewed the evolution of ML in disease diagnosis from the perspectives of algorithms, disease types, data types, and application scenarios. They identified explainable models and multi-modal data fusion as future core directions, providing a methodological benchmark for medical AI research. Pramanik and Sarker (2021) proposed an optimized framework for Parkinson’s disease detection based on high-dimensional small-sample voice data. Through multi-stage data preprocessing (normalization, multicollinearity elimination, dimensionality reduction) combined with ensemble learning strategies, they achieved an accuracy of 94.1% on a voice dataset, an 8% improvement over similar studies, and validated the robustness of random forest in imbalanced class scenarios. Almeida et al. (2019) proposed a multi-channel voice analysis framework. By comparing the performance differences between heart-shaped directional microphones (AC) and smartphone microphones (SP) in Lithuanian pronunciation tasks (sustained vowel /a/) and short sentence tasks, they verified that pronunciation task classification performed significantly better than speech tasks (AC accuracy 94.55% vs. SP 92.94%). Their multi-metric evaluation (AUC 0.87–0.92, EER 14.15–19.01%) revealed the impact of device type on Parkinson’s disease detection sensitivity, providing key parameters for optimizing mobile medical devices.

Compared to previous studies, this research made breakthroughs in preventing model overfitting. Previous studies often focused on larger datasets, but real-world scenarios are often limited by sample size and feature selection. This study used a small dataset with 195 voice records and employed various advanced machine learning algorithms to reduce the risk of overfitting and improve the model’s generalization ability. Additionally, SHAP methods were employed to assess model interpretability, providing strong support for understanding how machine learning models make predictions and facilitating their application in practical clinical environments.

From a practical standpoint, the proposed method is cost-effective compared to traditional diagnostic imaging or laboratory-based procedures. Voice recording can be collected using standard microphone devices or mobile phones, and the analysis process can be automated and completed in under a minute using pre-trained models. Furthermore, minimal training is required for healthcare personnel, as the interface for the system can be designed to be user-friendly and integrated into electronic health record systems. These features make the approach particularly suitable for primary care settings, remote screening, or telemedicine applications.

Furthermore, the promising results of this study suggest that the proposed voice-based detection framework may hold potential for broader clinical applications beyond Parkinson’s disease. Given that speech impairments are also prevalent in other neurodegenerative disorders such as Alzheimer’s disease and amyotrophic lateral sclerosis (ALS), future research could explore the adaptation of this methodology to assist in early screening and monitoring of these conditions. Integrating such non-invasive tools into the diagnostic pipeline may contribute to timely intervention and improved patient outcomes across a range of neurodegenerative diseases.

Despite offering a speech data-based PD prediction model, this study has several limitations. First, the dataset is relatively limited, which affects the model’s generalizability. Future research should expand the dataset to include more patients with diverse features, such as images and clinical indicators, to improve the model’s universality and robustness. Second, although various machine learning algorithms were evaluated, the sensitivity of these algorithms to noise and atypical samples needs further exploration. Moreover, the data collection was limited to a single language and speech feature; future research could consider incorporating other languages or speech features (such as emotional tone) to further improve diagnostic accuracy. Additionally, the UCI Parkinson’s Speech Dataset lacks detailed clinical metadata such as Hoehn and Yahr (H&Y) staging, UPDRS scores, medication status, and non-motor symptoms, which limits the model’s clinical interpretability. Specifically, we plan to integrate acoustic features with detailed clinical data, such as Hoehn and Yahr (H&Y) staging, UPDRS scores, medication usage, and non-motor symptoms. This integration will allow for more comprehensive patient stratification based on disease severity, treatment response, and symptom subtypes. Moreover, combining these clinical indicators with acoustic biomarkers can provide a deeper understanding of the relationship between vocal impairments and disease progression in Parkinson’s disease. Future research should also explore the potential of longitudinal datasets to track changes in speech features over time, offering dynamic insights into disease progression and treatment outcomes. By integrating this multimodal data, the clinical applicability and diagnostic accuracy of the model can be significantly enhanced, making it more effective for use in personalized healthcare settings.

## 5 Conclusion

This study demonstrates the potential of machine learning models based on speech data for the early detection of PD. Through feature selection, model construction, and SHAP analysis, we identified important speech features associated with PD and proved the superiority of models in prediction accuracy and clinical decision support. In the future, with the expansion of datasets and further optimization of models, this non-invasive diagnostic tool based on speech analysis is expected to become an important auxiliary method for early detection of PD, providing directions for disease management and personalized treatment.

## Data Availability

The original contributions presented in the study are included in the article/supplementary material, further inquiries can be directed to the corresponding authors.
